# Lycopene and kidney; future potential application

**Published:** 2015-05-14

**Authors:** Zahra Taheri, Mahin Ghafari, Masoud Amiri

**Affiliations:** ^1^Social Health Determinants Research Center, Shahrekord University of Medical Sciences, Shahrekord, Iran

**Keywords:** Lycopene, Antioxidant, Oxidative stress, Inflammation, Kidney disease

**Implication for health policy/practice/research/medical education:**

Lycopene is a lipid-soluble antioxidant and has been inversely associated with lipid peroxidation, including low-density lipoprotein oxidation and reduced oxidative stress and inflammation. In addition, lycopene has a variety of biological activities such as aging prevention, cancer prevention, anti inflammation, and oxidative. Many chronic diseases, such as cardiovas¬cular disease, cancer, diabetes and eye diseas¬es are the result of prolonged oxidative stress. Lycopene could be considered as the most effective antioxidant among the carotenoids. Lycopene is a potent antioxidant that offers protection for cellular damage due to reactive oxygen species.

## Introduction

Lycopene (C40H56), as a unsaturated red-pigmented linear carotenoid, has a molecular weight of 536.85 Da, and 11 conjugated and 2 non-conjugated double bonds; it is lipophilic and therefore more soluble in organic solvents (1-3). Lycopene is a lipid-soluble antioxidant and has been inversely associated with lipid peroxidation, comprising low-density lipoprotein oxidation and reduced oxidative stress and inflammation. In addition, lycopene can be considered as the most predominant carotenoid in plasma of human (4). Moreover, it is a carotenoid that is naturally found in tomatoes which acts as an antioxidant (5) and a potential chemo-preventive agent (6) with a singlet-oxygen and free radical scavenging capacity (7). A half-life of about 2–3 days can be considered for it when consumed. There is no official recommended amount for the daily intake of lycopene (8,9). Tomatoes could provide almost 85% of the lycopene in the diet as well as watermelon, guava, pink grapefruit, and rosehip (10).

Lycopene is one of more than 600 carotenoids were found in the nature with a variety of biological activities such as aging prevention, cancer prevention, anti‐inflammation, and oxidative (11). It contains a high level of antioxidant to help preventing different kinds of oxidative damages in tissues and cells. Lycopene has indeed received special attention due to the fact that it is a highly efficient antioxidant as well as having the function of singlet‐oxygen and free radical scavenging (6,12,13). Thus, lycopene, as a bioactive compound, may help prevention of chronic disease (14). Although lycopene is a potent antioxidant in vitro; however, its role in human health might be because of its bioactive metabolites.

It has been suggested that many noncommunicable and chronic diseases, such as cardiovascular disease, cancer, diabetes and eye diseases are the result of long standing oxidative stress. Antioxidants such as vitamin E, lycopene and tocopherols, may have an important role to play in protection against oxidative damage. Lycopene, as an effective antioxidant and a free radical scavenger has been demonstrated by several studies. Research has suggested that lycopene could reduce lipid peroxidation and atherogenesis in hemodialysis patients with chronic renal failure (15,16). In fact, increased production of oxidative stress markers and decreased concentrations of antioxidants could define oxidative stress resulting from cisplatin (17,18).

## Lycopene and kidney

Several studies have investigated the potential protective effect of lycopene on many diseases such as impaired cardiac and renal function (19), cisplatin-induced nephrotoxicity and oxidative stress in rat (7), chronic kidney disease (CKD) (20), renal cell carcinoma (21,22), mercury kidney damage (23), oxidative stress and inflammation in the kidney due to obesity (24), renal dysfunction and oxidative stress (25), development of diabetic nephropathy and ameliorates renal function via improving oxidative status through strong antioxidant properties and also lipid-lowering effect (26), cisplatin-induced nephrotoxicity in rats (18,27), lipid peroxidation and atherogenesis in hemodialysis individual (16), DNA damage (28) and nephrotoxicity and oxidative stress (29) induced by ochratoxin A, colistin-induced nephrotoxicity in mice (30), contrast medium-induced oxidative stress, inflammation, autophagy, and apoptosis in rat kidney (31), kidney tissue diseases (32).

Lycopene is a potent antioxidant which could supply the protection against cellular damage due to reactive oxygen species (33,34). Antioxidant vitamins and dietary constituents (such as, vitamin C, tocopherols, a-carotene and other carotenoids) could play a substantial role in protection against oxidative damage (35-37). In addition, while there is interest in the association of lycopene and some diseases such as cardiovascular disease, eye health and skin, the main part of research has focused on prostate cancer. Various epidemiological studies have detected that higher intakes of tomato and tomato product consumption are associated with a reduced risk of prostate cancer (11,38,39). In addition, lycopene as a potent antioxidant, may contribute to the lessening of oxidative stress in metabolic diseases (40) as well as protection against oxidation of lipids, proteins and DNA (19).

## Conclusion

Lycopene could be considered as the most effective antioxidant among the carotenoids. It has been reported to be able to attenuate oxidative stress and exert its anticancer effects both in vitro and in vivo (33). Humans and animals could earn lycopene from different sources specially dietary sources, because they cannot synthesize it (41). Lycopene is shown to reduce the risk of some chronic diseases including cancer (For example prostate cancer) and cardiovascular disorders. Lycopene and vitamin C have been shown to have an influence on the biomarkers of oxidative stress and inflammation. Low plasma levels of lycopene and analgesic consumption may increase more the risk of CKD. Lycopene not only can significantly decrease plasma total cholesterol, triglyceride and low density lipoprotein cholesterol levels and conversely increase high density lipoprotein cholesterol value (26). In fact, the favorable effects of lycopene is being as an anti-inflammatory, anti-autophagic, and antiapoptotic substance (31).

## Authors’ contribution

AT and MG prepared main draft and MA edited it. All authors read and signed the final manuscript.

## Conflicts of interest

The authors declared no competing interests.

## Ethical considerations

Ethical issues have been completely observed by the authors.

## Funding/Support

None.
